# Immediate effects of photobiomodulation on saliva production

**DOI:** 10.1590/2317-1782/20242023224en

**Published:** 2024-05-27

**Authors:** Amanda Rentero Gimenez do Amaral Silva, Lucas de Oliveira Cunha, Déborah Carollina Costa Silva, Vanessa Mouffron Novaes, Aline Mansueto Mourão, Laélia Cristina Caseiro Vicente

**Affiliations:** 1 Departamento de Fonoaudiologia, Universidade Federal de Minas Gerais – UFMG - Belo Horizonte (MG), Brasil.

**Keywords:** Salivary Glands, Saliva, Sialorrhea, Xerostomia, Low-level Light Therapy, Perception

## Abstract

**Purpose:**

To verify the immediate effects of photobiomodulation on the production of salivary flow and the correlation of demographic, anthropometric and medication use data.

**Methods:**

The study included 100 healthy individuals, aged between 18 and 76 years (mean 27.2 years), randomly split into an experimental group and a placebo group. Assessments of anthropometric measurements, self-perception of saliva production and sialometry were performed. Next, LASER irradiation was carried out at an infrared wavelength (808 nanometers) with 100 milliwatts (mw) of power at five intraoral points: on the sublingual glands and bilaterally on the submandibular and parotid glands, at doses of 9, 18 and 24 joules (J). Sialometry was repeated after each application. The control group received the same procedures with placebo equipment.

**Results:**

There was a statistical association in the self-perception of reduced saliva in the experimental group for the 24J dose and in sialometry and in the reduction in salivary flow for the 18J and 24J doses and an increase to 9J, in both groups. There was no association when comparing the experimental and placebo groups. Multiple multinomial regression analysis revealed that the reduction or increase in salivary flow is independent of demographic, anthropometric and medication use variables.

**Conclusion:**

The bioinhibitory action of photobiomodulation on healthy salivary glands occurred at a dose of 18J and 24J, while the biostimulant action happened at a dose of 9J, regardless of demographic, anthropometric variables and medication use. The self-perception of reduced salivary flow occurred at 24J.

## INTRODUCTION

Photobiomodulation is a therapeutic resource applying light energy to promote the modulation of biological processes, aiding in some treatments in various areas of health. Several effects are feasible to be achieved with low-intensity LASERs, including optimizing tissue repair, analgesia, improving lymphatic drainage and muscle condition, either by optimizing myofunctional training or by promoting relaxation^(1)^.

One subject that has been much discussed in the current literature is the effects of this resource on modulating salivary flow. Most studies on the subject have found positive effects of photobiomodulation in the treatment of xerostomia, especially after radiotherapy treatments in the head and neck region^(2-6)^.

In the speech-language therapy clinic, a major challenge is the management of saliva in patients with swallowing disorders, since excess saliva in the oral cavity harms stomatognathic functions, increases the risk of bronchoaspiration and worsens quality of life^(7,8)^. Speech-language therapists can help reduce this condition by providing guidance and swallowing therapy, including the use of some auxiliary therapeutic resources^(9,10)^. The aims of therapy are to improve the sensitivity, mobility and tone of the structures in the oral cavity in order to increase the frequency and efficiency of saliva swallowing.

Different clinical conditions may lead to disorders characterized by a lack, excess or accumulation of saliva. However, speech therapy has its limitations, especially for unresponsive patients with alterations resulting from neurological impairment. In these cases, there are invasive medical and drug approaches to controlling sialorrhea, including anticholinergic medication, botulinum toxin application, salivary gland radiotherapy and even surgery, all with their own pros and cons^(11-14)^.

Among the therapeutic resources that speech-language therapists can use to control saliva, photobiomodulation has gained prominence in clinical practice^(15)^. It is known that a biphasic dose-response has often been observed in various studies related to photobiomodulation, in which low levels of energy have an effect on tissue stimulation and repair, while higher levels show a reduction in biological activity and inhibitory action^(16)^. However, the literature search failed to find articles investigating the bioinhibitory action of photobiomodulation on healthy salivary glands.

Against this backdrop, the aims of this study were to assess the immediate effects of photobiomodulation on self-perception and salivary production in healthy individuals and to verify the correlation between anthropometric and demographic data and the use of medication and salivary production.

## METHODS

This is an experimental, blind, randomized study with a non-probabilistic, convenience sample, carried out at the Speech-language Therapy Observatory of the Federal University of Minas Gerais. The study was cleared by the institution's Research Ethics Committee under protocol number 3.662.623 and all participants signed an informed consent form.

Participants were recruited by invitation to students and staff from the Faculty of Medicine at the Federal University of Minas Gerais and people who were close to the researchers involved. All candidates who expressed an interest in taking part were subjected to a simplified clinical assessment, drawn up by the researchers themselves in order to determine whether they met the inclusion and exclusion criteria.

Adults of both sexes over the age of 18 who had preserved oral communication and cognitive skills, no history of neurological diseases or craniofacial deformities and no complaints related to saliva production or swallowing could take part in the study. The exclusion criteria were related to contraindications for photobiomodulation, such as the presence of photosensitivity, injury or infection at the application site, glaucoma or a history of tumor in the region to be irradiated.

The sample consisted of 100 subjects, who were randomly split into two groups with the same number of participants: Experimental Group (EG) and Placebo Group (PG).

Data was collected in two stages, which will be described below.

### Initial assessment

This stage consisted in the anthropometric measurements (height, weight and body mass index), collection of data on self-perception of salivary flow and medication use, as well as a quantitative assessment of saliva production was carried out.

To obtain the anthropometric data, the participants were instructed to stand barefoot on a platform, keeping their feet together and their head upright at a 90° angle. Height was measured in centimeters (cm) using a Personal Caprice Portable 2060 Sanny stadiometer. Body weight was measured in kilograms (kg) and Body Mass Index (BMI) in kilograms per square meter (Kg/m^2^), both obtained using the Tanita UM080 Fat and Water Monitor Scale.

Self-perception of salivary flow was assessed using a Likert scale. Participants marked the number on a scale between 0 and 10 that best represented their perception of the amount of saliva in their mouths at the time of the assessment. On this scale, 0 was considered to be no saliva, 5 the usual amount and 10 an increased amount.

The quantitative assessment of saliva was carried out using the Halitus sialometry kit, according to the manufacturer's instructions^(17)^. Salivation was stimulated by chewing on an unflavored latex tourniquet strip for three minutes. The participants were instructed, whenever they felt the urge to swallow, to deposit their saliva in a millimeter-sized saliva receiver tube and to keep chewing the tourniquet until the time was up. During the three minutes, the researcher monitored the participants' swallowing using the four-finger posture and cervical auscultation, in order to check for possible unconscious swallowing that could alter the results of the study. When the individual swallowed during the sialometry procedure, the time count was restarted.

After collection, three drops of dimethicone, an anti-foaming agent that allows salivary foam to precipitate quickly, were added to the container. The liquid was then mixed with a plastic spatula until all the foam was incorporated into the saliva and the volume of saliva produced during the three minutes of chewing could be seen in millimeters. To carry out the analysis, the volume of saliva produced in three minutes was divided by three to find the amount equivalent to one minute of salivation.

### Low-intensity LASER application

After the initial assessment, the low-intensity laser was applied. The equipment used was MMOPTICS, model Laser Duo, with 100 mW of power, continuous emission, output beam area equal to 3 square millimeters and power density equal to 3.33 W/cm^2^. For this study, we chose the infrared wavelength (808 nm) and the doses of 9 Joules (J), 18 J and 24 J. The application was done pointwise, with contact, at five intraoral points: towards the sublingual gland - with equipment directed towards the caruncles, towards the right and left submandibular glands - with a beam directed towards the floor of the mouth - and towards the parotid glands, also bilaterally, with equipment directed towards the upper retromolar region. The other dosimetry parameters are detailed in Chart 1.

**Chart 1:** Irradiation dosimetry parameters

Irradiation parameters	Application 1	Application 2	Application 3	Total application in each glandule
Output power (mW)	100 mW	100 mW	100 mW	N/R
Energy by point (J)	9	9	6	24
Time by point (s)	90	90	60	240
Application modality	Pointed with contact	Pointed with contact	Pointed with contact	N/R
Energy density	300 J/ in^2^ / sq in	300 J/ in^2^ / sq in	200 J/ in^2^ / sq in	800 J/ in^2^ / sq in
Power density	33.3 W/ in^2^ / sq in	33.3 W/ in^2^ / sq in	33.3 w/ in^2^ / sq in	N/R

Caption: mW = miliwatt; J = Joule; s = seconds; J/cm^2^ = Joule per square inch; W/cm^2^ = watt per square inch; N/R = nothing to report

The participants were randomly and blindly allocated to one of the following groups: placebo group (PG) (n=50) and experimental group (EG) (n=50) and underwent the following interventions:

- Application 1: Irradiation with 9 J of energy per point.
- Application 2: Irradiation with 9 J, totaling 18 J of accumulated energy in each gland.
- Application 3: Irradiation with 6 J, totaling 24 J of accumulated energy in each gland.

Between each of the irradiations and after the application, the self-perception assessment and sialometry procedures were repeated, carrying out the same procedures as the initial assessment. In the control group, the procedures were carried out in the same way as in the experimental group, but with placebo equipment lent by the manufacturer. The only difference between the two was the power output. In the placebo equipment, the manufacturer only has a red guide light, with an output power of less than 20 mW to guide the direction of the beam.

In accordance with the manufacturer's recommendations and the safety standards established by ANVISA for low-intensity LASER equipment, the researcher responsible for the application and the participants wore protective goggles throughout the procedure.

Regarding biosafety, the equipment was sanitized with 70% alcohol between each participant and wrapped in transparent plastic film. Participants were instructed to inform the researcher if they felt any discomfort during the procedures.

Statistical analysis was carried out using the IBM SPSS Statistics version 24 program. All the analyses were carried out by stratifying the experimental and placebo groups and the description of the data was presented in the form of observed frequency, percentage, median, mean and standard deviation. Pearson's chi-square test associated the groups (experimental and placebo) between the self-perceived saliva production and sialometry records at each stage and, when its assumptions were not met, Fisher's exact test was used. The chi-square test was used to compare the proportions of the immediate effects of photobiomodulation between the self-perceived saliva production and sialometry classifications (maintained, reduced or increased) at each dose and Fisher's test for the relationship between the drugs and sialometry, also at doses of 9, 18 and 24 joules in each group. Multiple multinomial regression using the backward variable selection method associated sialometry records with sociodemographic, anthropometric and medication variables. Finally, Spearman's correlation associated self-perception of saliva production with sialometry at each dose. The alpha level of significance used in all the analyses was 5%.

## RESULTS

In this study, 68.0% of the participants were female and 62.0% used medication. With regard to anthropometric measurements, the average height was 1.67 meters (± 0.09 meters), the average weight was 68.3 kilograms (± 16.4 kilograms) and the average BMI was 24.44 kg/cm^2^ (± 4.79 kg/cm^2^). The average body water content was 53.1% (± 7.1%) and body fat content 25.7% (± 9.9%) (Table 1).

**Table 1:** Description of participants' gender, use of medication and anthropometric variables

		Characteristics of the laser applied					
		Total		Experimental		Placebo	
		n	%	n	%	n	%
Sex	Men	32	32.0	16	32.0	16	32.0
	Women	68	68.0	34	68.0	34	68.0
Medicines	Yes	62	62.0	28	56.0	34	68.0
	No	38	38,.0	22	44.0	16	32.0
		Median	Mean (± SD)	Median	Mean (± SD)	Median	Mean (± SD)
Age (years)		23.0	27.2 (± 10.9)	22.5	26.7 (± 10.2)	24.0	27.7 (± 11.6)
Height (meters)		1.65	1.67 (± 0.09)	1.66	1.68 (± 0.09)	1.63	1.66 (± 0.09)
Weight (kg)		65.3	68.3 (± 16.4)	64.3	68.3 (± 16.2)	66.0	68.3 (± 16.8)
Body Mass Index (kg/cm^2^)		23.39	24.44 (± 4.79)	23.05	24.20 (± 4.99)	23.94	24.69 (± 4.61)
Body water rate (%)		52.8	53.1 (± 7.1)	53.3	53.0 (± 5.6)	52.0	53.2 (± 8.3)
Body fat rate (%)		25.5	25.7 (± 9.9)	23.3	24.4 (± 8.7)	27.4	27.1 (± 11.0)

Caption: n = number of participants; SD = standard deviation

Table 2 presents the responses obtained in each group for the different amounts of energy. It can be seen that the majority of participants in the experimental group reported a feeling of decreased saliva after 9J, although sialometry in this group, for the same energy, showed increased salivary production. For the doses of 18 J and 24 J, both groups also reported a feeling of decreased salivary flow, compatible with the sialometry findings. The intra-group analyses revealed that the self-perception of reduced saliva was statistically significant in the experimental group for the 24 J dose. In sialometry, there was a statistically significant reduction in salivary flow for the 18 J and 24 J doses and an increase for the 9 J dose in both groups, but to a greater extent in the experimental group (Table 2).

**Table 2:** Comparison of self-perception of saliva production and intra-group sialometry

			Photobiomodulation applied					
			Experimental			Placebo		
			n	%	P-value^*^	n	%	P-value*
Self-perception of saliva production after application	9 joules	Kept	12	24.0	0.151	11	22.0	0.249
		Reduced	23	46.0		19	38.0	
		Increased	15	30.0		20	40.0	
	18 joules	Kept	9	18.0	0.074	10	20.0	0.130
		Reduced	21	42.0		21	42.0	
		Increased	20	40.0		19	38.0	
	24 joules	Kept	11	22.0	**0.018**	10	20.0	0.056
		Reduced	26	52.0		24	48.0	
		Increased	13	26.0		16	32.0	
Sialometry after application	9 joules	Kept	6	12.0	**< 0.001**	6	12.0	**0.003**
		Reduced	14	28.0		19	38.0	
		Increased	30	60.0		25	50.0	
	18 joules	Kept	4	8.0	**0.001**	5	10.0	**0.001**
		Reduced	24	48.0		25	50.0	
		Increased	22	44.0		20	40.0	
	24 joules	Kept	4	8.0	**< 0.001**	5	10.0	**0.001**
		Reduced	28	56.0		25	50.0	
		Increased	18	36.0		20	40.0	

* Chi-square test for one sample; significant if p<0.050

When comparing the responses between the experimental and placebo groups, there was no statistically significant relationship between self-perception of saliva production and sialometry (Table 3).

**Table 3:** Comparison of self-perception of saliva production and intergroup sialometry

			Characteristics of the applied laser				P-value^*^
			Experimental		Placebo		
			n	%	n	%	
Self-perception of saliva production after application	9 joules	Kept	12	24.0	11	22.0	0.619
		Reduced	23	46.0	19	38.0	
		Increased	15	30.0	20	40.0	
	18 joules	Kept	9	18.0	10	20.0	0.999
		Reduced	21	42.0	21	42.0	
		Increased	20	40.0	19	38.0	
	24 joules	Kept	11	22.0	10	20.0	0.866
		Reduced	26	52.0	24	48.0	
		Increased	13	26.0	16	32.0	
Sialometry of the individual after application	9 joules	Kept	6	12.0	6	12.0	0.603
		Reduced	14	28.0	19	38.0	
		Increased	30	60.0	25	50.0	
	18 joules	Kept	4	8.0	5	10.0	0.915
		Reduced	24	48.0	25	50.0	
		Increased	22	44.0	20	40.0	
	24 joules	Kept	4	8.0	5	10.0	0.832
		Reduced	28	56.0	25	50.0	
		Increased	18	36.0	20	40.0	

* Pearson's chi-square test or Fisher's exact test; significant if p<0.050

With regard to the correlation between self-perception of saliva and sialometry data, a statistically significant correlation was observed for doses of 18 J and 24 J. For the former, there was a very weak positive correlation (rhô=0.282) and for the latter, there was a weak positive correlation (rhô=0.450)^(18)^ (Table 4).

**Table 4:** Correlation of self-perception of saliva production with sialometry

		Sialometry values					
		9 joules		18 joules		24 joules	
		Rhô	P-value*	Rhô	P-value^*^	Rhô	P-value*
Self-perception of saliva production	9 joules	-0.021	0.884				
	18 joules			0.282	**0.047**		
	24 joules					0.450	**0.001**

* Spearman correlation; significant if p<0.50

Caption: Rhô = coefficient

An analysis of the association between the use of medication and sialometry was carried out between the groups. The results showed that there was no significant association between contraceptive, antidepressant and hormone replacement drugs and sialometry in the experimental and placebo groups. As for the antihypertensive drug, there was a reduction in salivary flow in 100.0% of the participants in the experimental group after 9J irradiation. In those who didn't use the antihypertensive, there was a 23.3% reduction in salivary flow, which was statistically significant (p=0.039). There was no association in the other dosimetry measures.

Multiple multinomial regression analysis was carried out with all the demographic and anthropometric variables and the use of medication. Table 5 shows the results of the final regression model. In sialometry, the regression revealed no association between the explanatory variables and laser application in the experimental and placebo groups at any dose. Therefore, the reduction or increase in salivary flow was independent of demographic and anthropometric variables and the use of medication (Table 5).

**Table 5:** Multiple multinomial regression between sialometry and explanatory variables

Groups	Dependent variable - sialometry after application		Independent Variables	p-value^*^	OR	95% CI for OR	
						Inferior limit	Upper limit
Experimental	9J	Reduced	Medicines - yes	0.544	0.579	0.099	3.379
			Medicines - no	-	(1)	-	-
		Increased	Medicines - yes	0.206	0.434	0.119	1.582
			Medicines - no	-	(1)	-	-
	18J	Reduced	Age	0.220	1.192	0.900	1.578
		Increased	Age	0.456	1.112	0.842	1.468
	24J	Reduced	BMI	0.813	0.980	0.828	1.159
			Medicines - yes	0.758	0.739	0.108	5.068
			Medicines - no	-	(1)	-	-
		Increased	BMI	0.150	0.879	0.737	1.048
			Medicines - yes	0.553	1.728	0.283	10.535
			Medicines - no	-	(1)	-	-
Placebo	9J	Reduced	Sex - men	0.585	0.592	0.090	3.886
			Sex - women	-	(1)	-	-
			Medicines - yes	0.282	0.277	0.027	2.871
			Medicines - no	-	(1)	-	-
		Increased	Sex - men	0.225	0.318	0.050	2.022
			Sex - women	-	(1)	-	-
			Medicines - yes	0.586	0.522	0.050	5.432
			Medicines - no	-	(1)	-	-
	18J	Reduced	Body water rate	0.892	0.993	0.893	1.104
		Increased	Body water rate	0.094	0.893	0.783	1.019
	24J	Reduced	Age	0.501	1.077	0.867	1.338
			Weight	0.818	0.964	0.705	1.318
			BMI	0.829	0.910	0.386	2.143
			Body fat rate	0.357	1.153	0.851	1.561
			Sex - men	0.481	12.302	0.011	133.322
			Sex - women	-	(1)	-	-
		Increased	Age	0.578	1.064	0.855	1.325
			Weight	0.419	0.878	0.640	1.204
			BMI	0.607	1.255	0.528	2.983
			Body fat rate	0.537	1.100	0.813	1.488
			Sex - men	0.402	20.011	0.018	220.948
			Sex - women	-	(1)	-	-

Note: The reference category of the dependent variable is maintained. Variables included in the initial model - Sex, age, height, weight, body mass index, body water rate, body fat rate, medications

* Multiple multinomial regression with backward selection method

Caption: BMI = Body mass index; J = joules; OR - *Odds Ratio*; (1) reference category; significant if p<0.050

## DISCUSSION

This study found that photobiomodulation with low-intensity laser promoted changes in participants' self-perception and salivary production. The effects of low-intensity LASER on the salivary glands have aroused the interest of research groups and a systematic review revealed positive results in the treatment of patients complaining of xerostomia or hyposalivation^(6)^. Most of these studies, however, were carried out with individuals who had a disease whose pathophysiology or treatment had a direct impact on salivary gland function.

We failed to find until now other studies investigating the action of LASERs in reducing saliva production. For this reason, when choosing the energies to be tested in this study, the authors took into account those most commonly used to stimulate salivary flow. A systematic review revealed that photobiomodulation has a beneficial effect on salivary gland function in cases of xerostomia or hyposalivation^(19)^. Based on the principles of the Arndt-Schultz law, according to which low-intensity stimuli accelerate the activity of the body's cells until a plateau is reached and, from then on, increasing the intensity will progressively suppress the body's activity^(16)^, the present study decided to investigate the effects of 9 J, 18 J and 24 J.

In regard to the wavelength to be used to control salivary flow, we know that infrared light is poorly absorbed by superficial tissues and therefore reaches deeper tissues^(3,18,19)^. The effectiveness of red (660 to 685 nanometers) and infrared (780 to 905 nanometers) wavelengths has already been compared in patients complaining of xerostomia^(6)^, and better results were found for the infrared wavelength^(2,3,20-23)^, justifying its choice for this study.

Considering the points used and the form of application, the light should be applied perpendicular to the target structure^(24)^, but there is no consensus as to whether applications to the parotid and submandibular glands should be extra or intraoral. A study of patients with head and neck cancer performed only intraoral applications^(25)^. No studies were found comparing different photobiomodulation protocols in terms of application mode, including extra or intraoral application or number of irradiated points. In this study, we opted for intraoral applications only, in order to better target the salivary glands. However, this method requires the participant to keep their mouth open for a prolonged period, causing discomfort or tiredness, and this factor should be considered in therapeutic programs.

The findings evidenced that with the 9 J dose there was an increase in salivary production. This finding is in line with the results of another study, which also observed an increase in salivary flow with the 10J dose^(24)^. The literature on treating xerostomia or hyposalivation with photobiomodulation is vast, especially in head and neck cancer cases^(2-6)^. The results show good effects in the treatment with the therapeutic resource with low levels of light, causing a biostimulant effect on the salivary glands.

The bioinhibitory effect with LASERs on the salivary flow of patients with difficulty managing saliva is an aim of many clinicians. The causes of swallowing disorders with saliva are diverse and treatment is directed to improve the biomechanics of swallowing. However, inability to manage saliva can make treatment procedures difficult, and resources need to be used to suppress saliva production. This study revealed that doses of 18J and 24J were able to reduce salivary flow in healthy individuals and this effect was only perceived by the participants at the highest dose. The comparison between the experimental and placebo groups showed no differences. It is believed that the emission of light by the placebo equipment, albeit of low power, together with the fact that the participants kept their mouths open for so long, may have been the factors behind the lack of difference between the groups. It is important to note that, according to the literature, photobiomodulation has better effects on diseased or damaged cells and tissues and little impact on healthy cells^(26)^, which may also explain the similarity in the results presented by the groups.

With regard to the correlation between self-perception of saliva and sialometry data, there was a weak significant correlation for doses of 18 J and 24 J. This result indicates that it is important for clinicians to have more precise ways of assessing the effects of photobiomodulation in their practice than just self-perception.

As for the use of medication, there was only an association with antihypertensive medication for the 9J experimental group, in which there was a reduction in salivary flow in sialometry. A systematic review study revealed that there is a correlation between reduced flow and the use of antihypertensive drugs^(27)^. The correlation between the use of medication and saliva production after LASER application needs to be better investigated, as the population participating in this study was mostly made up of young people active in their activities of daily living, the use of medication was not frequent, and studies with a more representative sample are needed in this regard.

This study also found that there was no association between demographic and anthropometric variables and LASER application. No other articles were found comparing the correlation between these factors and the use of photobiomodulation. One of the hypotheses postulated that anthropometric data, such as BMI or body fat, could interfere with the penetration of the light beams. However, the results showed that for salivary flow these variables were not influenced by the dosimetry and length of light used in the study.

The present study quantitatively investigated the effects of photobiomodulation on salivary flow at higher doses than those described in the literature. It should be emphasized that there was a significant difference in sialometry at the three doses applied, which could help define dosimetry parameters for salivary flow control. Based on these results, studies are needed to assess the effects of photobiomodulation on salivary flow in individuals with difficulty managing saliva or complaining of sialorrhea. Both immediate effects and effects after regular applications need to be investigated, helping to create protocols and dosimetry parameters for pathological conditions and unresponsive patients.

## CONCLUSIONS

The bioinhibitory action of photobiomodulation on healthy salivary glands occurred at doses of 18J and 24J, while the biostimulant action at dose 9J was independent of demographic and anthropometric variables and the use of medication. Self-perception of reduced salivary flow occurred at 24J.
