# Supplementary material for: Immediate effects of photobiomodulation on saliva production
Source: Codas. 2024 May 27;36(3):e20230224. doi: 10.1590/2317-1782/20242023224en (PMC11166038; doi:10.1590/2317-1782/20242023224en)
Supplement: Translation

# Translation: Efeitos imediatos da fotobiomodulação na produção de saliva

**DOI:** 10.1590/2317-1782/20242023224pt

**Authors:** Amanda Rentero Gimenez do Amaral Silva, Lucas de Oliveira Cunha, Déborah Carollina Costa Silva, Vanessa Mouffron Novaes, Aline Mansueto Mourão, Laélia Cristina Caseiro Vicente

**Affiliations:** 1 Departamento de Fonoaudiologia, Universidade Federal de Minas Gerais – UFMG - Belo Horizonte (MG), Brasil.

**Keywords:** Glândulas Salivares, Saliva, Sialorreia, Xerostomia, Terapia com Luz de Baixa Intensidade, Percepção

## Abstract

**Objetivo:**

Verificar os efeitos imediatos da fotobiomodulação na produção do fluxo salivar e a correlação dos dados demográficos, antropométricos e de uso de medicamentos.

**Método:**

Participaram do estudo 100 indivíduos saudáveis, com idade entre 18 e 76 anos (média 27,2 anos), divididos de forma randomizada em grupo experimental e grupo placebo. Foram realizadas as avaliações das medidas antropométricas, autopercepção da produção de saliva e a sialometria. Na sequência, realizou-se a irradiação do LASER no comprimento de onda infravermelho (808 nanômetros) com 100 miliwatts (mw) de potência em cinco pontos intraorais: nas glândulas sublingual e bilateralmente nas submandibulares e parótidas, nas doses 9, 18 e 24 joules (J). A sialometria foi repetida após cada aplicação. O grupo controle recebeu os mesmos procedimentos com equipamento placebo.

**Resultados:**

Houve associação estatística na autopercepção de redução da saliva no grupo experimental para a dose de 24J e na sialometria e na redução do fluxo salivar para as doses 18J e 24J e aumento para 9J, em ambos os grupos. Não houve associação quando comparado entre os grupos experimental e placebo. A análise de regressão multinomial múltipla revelou que a redução ou o aumento do fluxo salivar independe das variáveis demográficas, antropométricas e uso de medicamentos.

**Conclusão:**

A ação bioinibitória da fotobiomodulação sobre as glândulas salivares saudáveis ocorreu em dose de 18J e 24J, já ação bioestimulante na dose 9J, independe das variáveis demográficas, antropométricas e uso de medicamentos. A autopercepção da redução do fluxo salivar ocorreu em 24J.

## INTRODUÇÃO

A fotobiomodulação é um recurso terapêutico que utiliza a energia luminosa para promover a modulação de processos biológicos, auxiliando em alguns tratamentos em diversas áreas da saúde. São diversos os efeitos possíveis de serem alcançados com o LASER de baixa intensidade, dentre eles a otimização do reparo tecidual, a analgesia, melhora da drenagem linfática e da condição muscular, seja otimizando o treinamento miofuncional ou propiciando seu relaxamento^(1)^.

Um tema bastante discutido na literatura atual são os efeitos desse recurso para modulação do fluxo salivar. Grande parte dos estudos acerca do tema encontraram efeitos positivos da fotobiomodulação no tratamento da xerostomia, principalmente após tratamentos de radioterapia em região de cabeça e pescoço^(2-6)^.

Na clínica fonoaudiológica, um grande desafio é o manejo da saliva em pacientes com transtorno de deglutição, uma vez que o excesso de saliva na cavidade oral traz prejuízos para as funções estomatognáticas, aumenta o risco de broncoaspiração e piora a qualidade de vida^(7,8)^. O fonoaudiólogo pode contribuir para redução dessa condição por meio de orientações e de terapia para deglutição, incluindo o uso de alguns recursos terapêuticos auxiliares^(9,10)^. Os objetivos da terapia visam melhorar a sensibilidade, mobilidade e tônus das estruturas da cavidade oral, a fim de aumentar a frequência e a eficiência das deglutições de saliva.

Diferentes condições clínicas podem levar o paciente a apresentar distúrbios caracterizados pela falta, excesso ou acúmulo de saliva. No entanto, a atuação fonoaudiológica possui limitações, especialmente junto aos pacientes pouco responsivos, com alterações decorrentes de comprometimentos neurológicos. Nesses casos, existem abordagens médicas invasivas e medicamentosas para o controle da sialorreia, dentre elas, medicações anticolinérgicas, aplicação de toxina botulínica, radioterapia em glândulas salivares e até mesmo abordagem cirúrgica, todas essas com prós e contras^(11-14)^.

Dentre os recursos terapêuticos que o fonoaudiólogo pode utilizar para o controle da saliva, a fotobiomodulação tem ganhado destaque na prática clínica^(15)^. Sabe-se que uma resposta de dose bifásica tem sido frequentemente observada em diversos estudos relacionados à fotobiomodulação, nos quais baixos níveis de energia têm efeito na estimulação e reparação de tecidos, enquanto níveis mais altos demonstram redução da atividade biológica e ação inibitória ^((16))^. No entanto, não foi encontrado na literatura trabalho que investigasse a ação bioinibitória da fotobiomodulação sobre glândulas salivares saudáveis.

Diante disso, os objetivos deste estudo foram avaliar os efeitos imediatos da fotobiomodulação sobre a autopercepção e produção salivar de indivíduos saudáveis e verificar a correlação dos dados antropométricos, demográficos e do uso de medicamentos com a produção de saliva.

## MÉTODO

O presente trabalho trata-se de um estudo experimental, cego, randomizado, com amostra não probabilística, por conveniência, realizado no Observatório de Fonoaudiologia da Universidade Federal de Minas Gerais. O estudo foi aprovado pelo Comitê de Ética em Pesquisa da instituição sob protocolo número 3.662.623 e todos os participantes assinaram o Termo de Consentimento Livre e Esclarecido.

O recrutamento dos participantes foi realizado por meio de convites aos alunos e funcionários da Faculdade de Medicina da Universidade Federal de Minas Gerais e às pessoas do convívio dos pesquisadores envolvidos. Todos os candidatos que manifestaram interesse em participar foram submetidos à avaliação clínica simplificada, elaborada pelos próprios pesquisadores a fim de definir o enquadramento nos critérios de inclusão e exclusão.

Poderiam participar do estudo indivíduos adultos, de ambos os sexos, com idade superior a 18 anos e que apresentassem capacidade de comunicação oral e cognitivo preservados, ausência de histórico de doenças neurológicas ou de deformidades craniofaciais e que não tivessem queixas relacionadas à produção de saliva ou deglutição. Já os critérios de exclusão correlacionaram-se com às contraindicações para fotobiomodulação, como presença de fotossensibilidade, lesão ou infecção no local de aplicação, glaucoma ou histórico de tumor na região a ser irradiada.

A amostra foi constituída por 100 sujeitos, que foram divididos, de modo randomizado, em dois grupos com o mesmo número de participantes: Grupo Experimental (GE) e Grupo Placebo (GP).

A coleta dos dados foi realizada em duas etapas, que serão descritas a seguir.

### Avaliação inicial

Nesta etapa, foram coletadas as medidas antropométricas (altura, peso e índice de massa corporal), dados sobre a autopercepção do fluxo salivar e do uso de medicamentos e foi realizada a avaliação quantitativa da produção de saliva.

Para obtenção dos dados antropométricos, o participante foi orientado a permanecer de pé e descalço sobre um tablado, mantendo os pés juntos e a cabeça ereta em um ângulo de 90°. A altura foi medida em centímetros (cm) utilizando-se um estadiômetro da marca e modelo Personal Caprice Portátil 2060 Sanny. O peso corporal foi medido em quilogramas (kg) e o Índice de Massa Corporal - IMC em quilogramas por metro quadrado (Kg/m^2^), ambos obtidos por meio da Balança Monitor de Gordura e Água UM080- Tanita.

A avaliação da autopercepção do fluxo salivar foi realizada por meio da escala Likert. Os participantes assinalaram, na escala graduada de 0 a 10, o número que melhor representasse a percepção sobre a quantidade de saliva presente na boca no momento da avaliação. Nessa escala, considerou-se 0 como nenhuma saliva, 5 a quantidade habitual e 10 uma quantidade aumentada.

A avaliação quantitativa da saliva foi feita utilizando-se o kit de sialometria Halitus, conforme orientação do fabricante^(17)^. A salivação foi estimulada por meio da mastigação de uma tira de garrote de látex sem sabor, por três minutos. Os participantes foram orientados a, sempre que sentissem vontade de deglutir, depositar a saliva em um tubo receptor de saliva milimetrado e permanecerem mastigando o garrote até completar o tempo. Durante os três minutos, o pesquisador monitorou a deglutição dos participantes por meio da postura de quatro dedos e ausculta cervical, a fim de verificar possíveis deglutições inconscientes que pudessem alterar os resultados do estudo. Quando o indivíduo deglutia durante procedimento de sialometria, a contagem do tempo era reiniciada.

Após a coleta, eram acrescidas no recipiente três gotas de dimeticona, um agente antiespumante que permite uma rápida precipitação da espuma salivar. Em seguida, o líquido era misturado com espátula de plástico até que toda a espuma estivesse incorporada à saliva e fosse possível visualizar, em milímetros, o volume de saliva produzido nos três minutos de mastigação. Para realizar a análise, o volume de saliva produzido em três minutos foi dividido por três, com a finalidade de encontrar a quantidade equivalente a um minuto de salivação.

### Aplicação do LASER de baixa intensidade

Após a avaliação inicial, realizou-se a aplicação do LASER de baixa intensidade. O equipamento utilizado foi da marca MMOPTICS, modelo Laser Duo, com 100 mW de potência, emissão contínua, área do feixe de saída igual a 3 milímetros quadrados e densidade de potência igual a 3,33 W/cm^2^. Para esse estudo, optou-se pelo comprimento de onda infravermelho (808 nm) e as doses de 9 Joules (J), 18 J e 24 J. A aplicação foi feita de modo pontual, com contato, em cinco pontos intraorais: em direção à glândula sublingual – com equipamento direcionado para as carúnculas, em direção às submandibulares direita e esquerda – com feixe direcionado para o assoalho da boca - e em parótidas, também bilateralmente, com equipamento direcionado para a região retromolar superior. Os demais parâmetros dosimétricos estão detalhados no Quadro 1.

**Quadro 1:** Parâmetros dosimétricos de irradiação

Parâmetros de irradiação	Aplicação 1	Aplicação 2	Aplicação 3	Total da aplicação em cada glândula
Potência de saída (mW)	100 mW	100 mW	100 mW	N/A
Energia por ponto (J)	9	9	6	24
Tempo por ponto (s)	90	90	60	240
Forma de aplicação	Pontual com contato	Pontual com contato	Pontual com contato	N/A
Densidade de energia	300 J/cm^2^	300 J/cm^2^	200 J/cm^2^	800 J/cm2
Densidade de potência	33,3 W/cm^2^	33,3 W/cm^2^	33,3 w/cm^2^	N/A

Legenda: mW = miliwatt; J = Joule; s = segundos; J/cm^2^ = Joule por centímetro quadrado; W/cm^2^ = watt por centímetro quadrado; N/A = nada a informar

Os participantes foram alocados, de maneira randomizada e cega, em um dos seguintes grupos: grupo placebo (GP) (n=50) e grupo experimental (GE) (n=50) e foram submetidos às seguintes intervenções:

- Aplicação 1: Irradiação com 9 J de energia por ponto.
- Aplicação 2: Irradiação com 9 J, totalizando 18 J de energia acumulada em cada glândula.
- Aplicação 3: Irradiação com 6 J, totalizando 24 J de energia acumulada em cada glândula.

Entre cada uma das irradiações e após a aplicação, os procedimentos de avaliação da autopercepção e de sialometria foram repetidos, realizando os mesmos procedimentos da avaliação inicial. No grupo controle, os procedimentos foram realizados de maneira idêntica ao realizado no grupo experimental, porém com equipamento placebo, emprestado pela empresa fabricante. A única diferença entre ambos era a potência de saída. No equipamento placebo o fabricante mantém apenas uma luz guia vermelha, com potência de saída inferior a 20 mW para orientar no direcionamento do feixe.

Atendendo às recomendações do fabricante e às normas de segurança estabelecidas pela ANVISA para equipamentos de LASER de baixa intensidade, o pesquisador responsável pela aplicação e os participantes usaram óculos de proteção durante todo o procedimento.

No que diz respeito à biossegurança, os equipamentos foram higienizados com álcool 70% entre um participante e outro e envoltos em filme plástico transparente. Os participantes foram orientados a avisar ao pesquisador caso sentissem qualquer desconforto durante a realização dos procedimentos.

A análise estatística foi feita utilizando-se o programa IBM SPSS *Statistics version* 24. Todas as análises foram realizadas estratificando os grupos experimental e placebo e a descrição dos dados foi apresentada na forma de frequência observada, porcentagem, mediana, média e desvio padrão. O teste do qui-quadrado de Pearson associou os grupos (experimental e placebo) entre os registros de autopercepção de produção de saliva e sialometria em cada etapa e, quando este não obteve suas premissas atendidas, utilizou-se o teste Exato de Fisher. O teste do qui-quadrado foi realizado para comparar as proporções dos efeitos imediatos da fotobiomodulação entre as classificações da autopercepção de produção de saliva e sialometria (manteve, reduziu ou aumentou) em cada dose e o de Fisher para a relação dos medicamentos com a sialometria, também nas doses de 9, 18 e 24 joules em cada grupo. A regressão multinomial múltipla com o método de seleção de variáveis *backward* associou os registros de sialometria com as variáveis sociodemográficas, antropométricas e de medicamentos. Por fim, a correlação de Spearman associou a autopercepção de produção de saliva com a sialometria em cada dose. O nível alfa de significância utilizado em todas as análises foi de 5%.

## RESULTADOS

No presente estudo, 68,0% dos participantes eram do sexo feminino e 62,0% utilizavam medicamentos. Em relação às medidas antropométricas, a estatura média foi 1,67 metros (± 0,09 metros), o peso médio foi 68,3 quilogramas (± 16,4 quilogramas) e o IMC médio foi 24,44 kg/cm^2^ (± 4,79 kg/cm^2^). Já a taxa corporal de água média foi de 53,1% (± 7,1%) e a de gordura 25,7% (± 9,9%) (Tabela 1).

**Tabela 1:** Descrição do sexo, uso de medicamentos e variáveis antropométicas dos participantes

		Característica do laser aplicado					
		Total		Experimental		Placebo	
		n	%	n	%	n	%
Sexo	Masculino	32	32,0	16	32,0	16	32,0
	Feminino	68	68,0	34	68,0	34	68,0
Medicamentos	Sim	62	62,0	28	56,0	34	68,0
	Não	38	38,0	22	44,0	16	32,0
		Mediana	Média (± DP)	Mediana	Média (± DP)	Mediana	Média (± DP)
Idade (anos)		23,0	27,2 (± 10,9)	22,5	26,7 (± 10,2)	24,0	27,7 (± 11,6)
Estatura (metros)		1,65	1,67 (± 0,09)	1,66	1,68 (± 0,09)	1,63	1,66 (± 0,09)
Peso (quilogramas)		65,3	68,3 (± 16,4)	64,3	68,3 (± 16,2)	66,0	68,3 (± 16,8)
Índice de Massa Corporal (kg/cm^2^)		23,39	24,44 (± 4,79)	23,05	24,20 (± 4,99)	23,94	24,69 (± 4,61)
Taxa corporal de água (%)		52,8	53,1 (± 7,1)	53,3	53,0 (± 5,6)	52,0	53,2 (± 8,3)
Taxa corporal de gordura (%)		25,5	25,7 (± 9.9)	23,3	24,4 (± 8,7)	27,4	27,1 (± 11,0)

Legenda: n = número de participantes; DP = desvio padrão

Na Tabela 2 estão descritas as respostas obtidas em cada grupo, para as diferentes quantidades de energia. É possível perceber que a maioria dos participantes do grupo experimental referiu a sensação de diminuição da saliva após 9J, embora a sialometria neste grupo, para essa mesma energia, tenha demonstrado aumento da produção salivar. Para as doses de 18 J e 24 J, ambos os grupos também referiram sensação de diminuição do fluxo salivar, compatível com os achados da sialometria. As análises obtidas intragrupos revelaram que a autopercepção de redução da saliva foi estatisticamente significativa no grupo experimental para a dose de 24 J. Na sialometria, verificou-se redução estatisticamente significativa do fluxo salivar para as doses 18J e 24J e aumento para 9J, em ambos os grupos, porém com maior proporção no grupo experimental (Tabela 2).

**Tabela 2:** Comparação da autopercepção da produção de saliva e da sialometria intragrupos

			Fotobiomodulação aplicado					
			Experimental			Placebo		
			n	%	Valor p^*^	n	%	Valor p*
Autopercepção da produção de saliva após a aplicação	9 joules	Manteve	12	24,0	0,151	11	22,0	0,249
		Reduziu	23	46,0		19	38,0	
		Aumentou	15	30,0		20	40,0	
	18 joules	Manteve	9	18,0	0,074	10	20,0	0,130
		Reduziu	21	42,0		21	42,0	
		Aumentou	20	40,0		19	38,0	
	24 joules	Manteve	11	22,0	**0,018**	10	20,0	0,056
		Reduziu	26	52,0		24	48,0	
		Aumentou	13	26,0		16	32,0	
Sialometria do indivíduo após a aplicação	9 joules	Manteve	6	12,0	**< 0,001**	6	12,0	**0,003**
		Reduziu	14	28,0		19	38,0	
		Aumentou	30	60,0		25	50,0	
	18 joules	Manteve	4	8,0	**0,001**	5	10,0	**0,001**
		Reduziu	24	48,0		25	50,0	
		Aumentou	22	44,0		20	40,0	
	24 joules	Manteve	4	8,0	**< 0,001**	5	10,0	**0,001**
		Reduziu	28	56,0		25	50,0	
		Aumentou	18	36,0		20	40,0	

* Teste do qui-quadrado para uma amostra; significativo se p<0,050

Quando comparadas as respostas entre os grupos experimental e placebo, não houve relação estatisticamente significativa na autopercepção da produção da saliva e na sialometria (Tabela 3).

**Tabela 3:** Comparação da autopercepção da produção de saliva e a sialometria intergrupos

			Característica do laser aplicado				Valor p*
			Experimental		Placebo		
			n	%	n	%	
Autopercepção da produção de saliva após a aplicação	9 joules	Manteve	12	24,0	11	22,0	0,619
		Reduziu	23	46,0	19	38,0	
		Aumentou	15	30,0	20	40,0	
	18 joules	Manteve	9	18,0	10	20,0	0,999
		Reduziu	21	42,0	21	42,0	
		Aumentou	20	40,0	19	38,0	
	24 joules	Manteve	11	22,0	10	20,0	0,866
		Reduziu	26	52,0	24	48,0	
		Aumentou	13	26,0	16	32,0	
Sialometria do indivíduo após a aplicação	9 joules	Manteve	6	12,0	6	12,0	0,603
		Reduziu	14	28,0	19	38,0	
		Aumentou	30	60,0	25	50,0	
	18 joules	Manteve	4	8,0	5	10,0	0,915
		Reduziu	24	48,0	25	50,0	
		Aumentou	22	44,0	20	40,0	
	24 joules	Manteve	4	8,0	5	10,0	0,832
		Reduziu	28	56,0	25	50,0	
		Aumentou	18	36,0	20	40,0	

* Teste do qui-quadrado de Pearson ou Exato de Fisher; significativo se p<0,050

No que diz respeito à correlação entre a autopercepção de saliva e os dados da sialometria, observou-se correlação estatisticamente significativa para as doses de 18 J e 24 J. Para a primeira, houve correlação positiva muito fraca (rhô=0,282) e para a segunda, houve correlação positiva fraca (rhô=0,450)^(18)^ (Tabela 4).

**Tabela 4:** Correlação da autopercepção da produção de saliva com a sialometria

		Sialometria do indivíduo					
		9 joules		18 joules		24 joules	
		Rhô	Valor p^*^	Rhô	Valor p*	Rhô	Valor p*
Autopercepção da produção de saliva	9 joules	-0,021	0,884				
	18 joules			0,282	**0,047**		
	24 joules					0,450	**0,001**

* Correlação de Spearman; significativo se p<0,50

Legenda: Rhô = coeficiente

Foi realizada análise de associação entre o uso dos medicamentos e a sialometria entre os grupos. Os resultados revelaram que não houve associação significativa dos medicamentos anticoncepcionais, antidepressivos e reposição hormonal com a sialometria nos grupos experimental e placebo. Já para o medicamento anti-hipertensivo houve redução do fluxo salivar em 100,0% dos participantes do grupo experimental após a irradiação com 9J. Já em quem não usou o anti-hipertensivo, ocorreu redução do fluxo salivar em 23,3%, sendo estatisticamente significante (p=0,039). Nas demais dosimetrias não houve associação.

A análise de regressão multinomial múltipla foi realizada com todas as variáveis demográficas, antropométricas e uso de medicamentos. Na Tabela 5 são apresentados os resultados do modelo final da regressão. Na sialometria, a regressão revelou que não há associação entre as variáveis explicativas e aplicação do LASER nos grupos experimental e placebo em nenhuma dose. Portanto, a redução ou o aumento do fluxo salivar independe das variáveis demográficas, antropométricas e uso de medicamentos (Tabela 5).

**Tabela 5:** Regressão multinomial múltipla entre a sialometria e as variáveis explicativas

Grupos	Variável dependente - sialometria após a aplicação		Variáveis independentes	Valor p^*^	OR	IC de 95% para OR	
						Limite inferior	Limite superior
Experimental	9J	Reduziu	Medicamentos - sim	0,544	0,579	0,099	3,379
			Medicamentos - não	-	(1)	-	-
		Aumentou	Medicamentos - sim	0,206	0,434	0,119	1,582
			Medicamentos - não	-	(1)	-	-
	18J	Reduziu	Idade	0,220	1,192	0,900	1,578
		Aumentou	Idade	0,456	1,112	0,842	1,468
	24J	Reduziu	IMC	0,813	0,980	0,828	1,159
			Medicamentos - sim	0,758	0,739	0,108	5,068
			Medicamentos - não	-	(1)	-	-
		Aumentou	IMC	0,150	0,879	0,737	1,048
			Medicamentos - sim	0,553	1,728	0,283	10,535
			Medicamentos - não	-	(1)	-	-
Placebo	9J	Reduziu	Sexo - masculino	0,585	0,592	0,090	3,886
			Sexo - feminino	-	(1)	-	-
			Medicamentos - sim	0,282	0,277	0,027	2,871
			Medicamentos - não	-	(1)	-	-
		Aumentou	Sexo - masculino	0,225	0,318	0,050	2,022
			Sexo - feminino	-	(1)	-	-
			Medicamentos - sim	0,586	0,522	0,050	5,432
			Medicamentos - não	-	(1)	-	-
	18J	Reduziu	Taxa corporal de água	0,892	0,993	0,893	1,104
		Aumentou	Taxa corporal de água	0,094	0,893	0,783	1,019
	24J	Reduziu	Idade do indivíduo	0,501	1,077	0,867	1,338
			Peso do indivíduo	0,818	0,964	0,705	1,318
			IMC	0,829	0,910	0,386	2,143
			Taxa corporal de gordura	0,357	1,153	0,851	1,561
			Sexo - masculino	0,481	12,302	0,011	133,322
			Sexo - feminino	-	(1)	-	-
		Aumentou	Idade do indivíduo	0,578	1,064	0,855	1,325
			Peso do indivíduo	0,419	0,878	0,640	1,204
			IMC	0,607	1,255	0,528	2,983
			Taxa corporal de gordura	0,537	1,100	0,813	1,488
			Sexo - masculino	0,402	20,011	0,018	220,948
			Sexo - feminino	-	(1)	-	-

Nota: A categoria de referência da variável dependente é a manteve. Variáveis incluídas no modelo inicial - Sexo, idade, estatura, peso, índice de massa corporal, taxa corporal de água, taxa corporal de gordura, medicamentos

* Regressão multinomial múltipla com método de seleção *backward*

Legenda: IMC = Índice de Massa Corporal; J = joules; OR - *Odds Ratio*; (1) categoria de referência; significativo se p<0,050

## DISCUSSÃO

Observou-se neste estudo que a fotobiomodulação com LASER de baixa intensidade promoveu mudanças na autopercepção e na produção salivar dos participantes. Os efeitos do LASER de baixa intensidade nas glândulas salivares vêm despertando o interesse de grupos de pesquisas e uma revisão sistemática revelou resultados positivos no tratamento de pacientes com queixa de xerostomia ou hipossalivação^(6)^. Grande parte desses trabalhos, no entanto, foram realizados com indivíduos que apresentavam alguma doença cuja fisiopatologia ou tratamento promoviam impactos diretos na função das glândulas salivares.

Não se tem conhecimento, até o momento, de trabalhos realizados com o objetivo de investigar a ação do LASER na redução da produção de saliva. Por esse motivo, para escolha das energias a serem testadas neste estudo os autores levaram em consideração aquelas mais utilizadas para estímulo do fluxo salivar. Uma revisão sistemática revelou que a fotobiomodulação têm efeito benéfico na função das glândulas salivares em casos de xerostomia ou hipossalivação^(19)^. A partir daí, baseando-se nos princípios da lei de Arndt-Schultz, segundo a qual estímulos de baixa intensidade aceleram a atividade das células do organismo até que um platô é alcançado e, a partir daí, o aumento da intensidade irá, de maneira progressiva, suprimir a atividade do organismo^(16)^, para o presente estudo optou-se por investigar os efeitos de 9 J, 18 J e 24 J.

Com relação ao comprimento de onda a ser utilizado para controle do fluxo salivar, sabemos que a luz infravermelha é pouco absorvida por tecidos superficiais e, com isso, alcança tecidos mais profundos^(3,18,19)^. A eficácia dos comprimentos de onda vermelho (660 a 685 nanômetros) e infravermelho (780 a 905 nanômetros) já foi comparada em pacientes com queixa de xerostomia^(6)^, e foram encontrados melhores resultados para o comprimento de onda infravermelho^(2,3,20-23)^, o que justifica sua escolha para este estudo.

No que diz respeito aos pontos utilizados e à forma de aplicação, a luz deve ser aplicada de forma perpendicular à estrutura alvo^(24)^, no entanto não há consenso se as aplicações nas glândulas parótidas e submandibulares devem ser extras ou intraorais. Estudo com pacientes com câncer de cabeça e pescoço realizou aplicações apenas intraorais^(25)^. Não foram encontrados estudos que comparassem diferentes protocolos de fotobiomodulação no que tange ao modo de aplicação, incluindo aplicação extra ou intraoral ou número de pontos irradiados. Neste estudo, optou-se por aplicações apenas intraorais, a fim de atingir melhor as glândulas salivares. Todavia, essa forma faz com que o participante mantenha a boca aberta por período prolongado, causando desconforto ou cansaço, devendo esse fator ser considerado em programas terapêuticos.

Quanto aos achados, observou-se que com a dose de 9 J ocorreu aumento da produção salivar. Tal achado está de acordo com os resultados encontrados em outro estudo, que também observou aumento do fluxo salivar com a dose de 10J^(24)^. A literatura para tratar a xerostomia ou a hipossalivação por meio da fotobiomodulação é vasta, principalmente nos casos oncológicos de cabeça e pescoço^(2-6)^. Os resultados demonstram bons efeitos no tratamento com o recurso terapêutico com baixos níveis de luz, causando efeito bioestimulante nas glândulas salivares.

O efeito bioinibitório com LASER no fluxo salivar de pacientes com dificuldade no manejo da saliva é um anseio de muitos clínicos. As causas dos transtornos de deglutição com a saliva são diversas e o tratamento visa melhorar a biomecânica da deglutição. Contudo, a incapacidade no manejo da saliva pode dificultar os procedimentos de tratamento, sendo necessários utilizar recursos para suprimir a produção de saliva. No presente estudo revelou que doses de 18J e 24J foram capazes de reduzir o fluxo salivar em indivíduos saudáveis e esse efeito só foi percebido pelos participantes na dose mais elevada. A comparação entre os grupos experimental e placebo não indicou diferenças. Acredita-se que a emissão de luz pelo equipamento placebo, embora de baixa potência, somado ao fato de os participantes permanecerem com a boca aberta por tanto tempo podem ter sido os fatores da não diferença entre os grupos. É importante ressaltar que, de acordo com a literatura, a fotobiomodulação tem melhores efeitos em células e tecidos doentes ou danificados e pouco impacto sobre células saudáveis ^((26))^, o que também pode justificar a semelhança nos resultados apresentados pelos grupos.

No que diz respeito à correlação entre a autopercepção de saliva e os dados da sialometria, houve correlação significativa fraca para as doses de 18J e 24 J. Esse resultado indica que é importante que o clínico tenha formas mais precisas de avaliar os efeitos da fotobiomodulação em sua prática que não seja somente o relato da autopercepção.

Quanto ao uso de medicamentos, verificou-se associação apenas com medicamento anti-hipertensivo para o grupo experimental em 9J em que houve redução do fluxo salivar na sialometria. Um estudo de revisão sistemática revelou que há correlação entre a diminuição do fluxo com o uso de antihipertensivo^(27)^. A correlação do uso de medicamento e a produção de saliva após aplicação do LASER precisa ser melhor investigada, como a população participante deste estudo era em sua maioria composta por jovens ativos em suas atividades de vida diária, o uso de medicamentos não foi frequente, sendo necessários estudos com amostra mais representativa nesse quesito.

No presente estudo, verificou-se também que não houve associação entre as variáveis demográficas e antropométricas com a aplicação do LASER. Não foram encontrados outros artigos que comparassem a correlação destes fatores no uso da fotobiomodulação. Uma das hipóteses era que os dados antropométricos, como IMC ou a taxa corporal de gordura, pudessem interferir na penetração dos feixes de luz. Todavia os resultados revelaram que para o fluxo salivar essas variáveis não sofreram influência da dosimetria e do comprimento de luz empregados no estudo.

Este estudo investigou de modo quantitativo os efeitos da fotobiomodulação no fluxo salivar em doses mais altas do que as descritas na literatura. É necessário ressaltar que, nas três doses aplicadas, houve significância na sialometria, o que pode contribuir para definição de parâmetros de dosimetria no controle do fluxo salivar. A partir desses resultados, são necessários estudos que avaliem os efeitos da fotobiomodulação no fluxo salivar de indivíduos com dificuldade no manejo da saliva ou com queixa de sialorreia. É preciso investigar tanto efeitos imediatos como após aplicações regulares, auxiliando na criação de protocolos e parâmetros dosimétricos para quadros patológicos e pacientes pouco responsivos.

## CONCLUSÕES

A ação bioinibitória da fotobiomodulação sobre as glândulas salivares saudáveis ocorreu em dose de 18J e 24J, já ação bioestimulante na dose 9J, independe das variáveis demográficas, antropométricas e uso de medicamentos. A autopercepção da redução do fluxo salivar ocorreu em 24J.
